# Periodontal regeneration with Bio-Oss for intrabony defect management: A case report

**DOI:** 10.6026/973206300214659

**Published:** 2025-12-15

**Authors:** K. Lakshmi Priya, Uma Subbiah, Sarumathi D, Sruthika G, Pavithra D, Jaideep Mahendra

**Affiliations:** 1 Department of Periodontics, Meenakshi Ammal Dental College and Hospital, Meenakshi Academy of Higher Education and Research (MAHER), Chennai, Tamil Nadu, India

**Keywords:** Chronic periodontitis, intrabony defect, guided tissue regeneration, bone graft

## Abstract

The goal of periodontal therapy is to preserve and regenerate the supporting structures of teeth affected by periodontitis. This report presents the management of an intrabony defect associated with teeth 16 and 17 in a 37-year-old female patient using demineralized bovine bone mineral (Bio-Oss®; Geistlich Pharma AG, Switzerland) and a GTR membrane (Bio-Gide®; Geistlich Pharma AG, Switzerland). Following initial non-surgical therapy, open flap debridement with regenerative treatment was performed. Clinical and radiographic follow-ups at one, three, and six months demonstrated a marked reduction in pocket depth and radiographic bone fill, indicating successful periodontal regeneration. Thus, we show the effectiveness of xenograft-based regenerative therapy in localized chronic periodontitis.

## Background:

Chronic periodontitis is a multifactorial inflammatory disease that leads to progressive loss of periodontal attachment and alveolar bone. The destruction of supporting tissues results from a complex interaction between pathogenic microorganisms and the host immune response. Vertical or intrabony defects represent a unique challenge in periodontal therapy because of their limited capacity for spontaneous bone regeneration. The advent of regenerative materials and biologically based techniques has significantly improved clinical outcomes by facilitating selective repopulation of the defect area with periodontal ligament cells capable of regenerating cementum, alveolar bone, and periodontal fibers [1]. Among the wide range of regenerative materials, deproteinized bovine bone mineral (Bio-Oss) is one of the most extensively used xenografts. It exhibits excellent biocompatibility, high porosity, and structural similarity to human cancellous bone, allowing osteoconduction and gradual replacement by new bone. It used in combination with a collagen membrane, guided tissue regeneration (GTR) promotes compartmentalized healing, excluding epithelial cells and gingival fibroblasts that could otherwise interfere with bone regeneration [2]. Therefore, it is of interest to report describes the management of a localized intrabony defect in the maxillary posterior region using Bio-Oss in combination with a collagen membrane, with favorable clinical and radiographic outcomes.

## Case history:

A 37-year-old female reported to the Department of Periodontics on 24 December 2024 with a chief complaint of bleeding gums and occasional food lodgment in the upper right posterior region for the past six months. The patient was systemically healthy and a non-smoker, with no significant medical or family history of periodontal disease.

## Clinical examination:

Intraoral examination revealed fair oral hygiene with localized gingival inflammation in relation to teeth 16 and 17, along with bleeding on probing. Root canal treatment was done in relation to 17, and the crown was found to be ill-fitting. The probing pocket depth measured 7 mm on the distal aspect of tooth 16 and the mesial aspect of tooth 17. Tooth 16 exhibited Grade I mobility, while 17 remained stable. A Grade I furcation involvement was noted on the buccal aspect of 16.

## Radiographic findings:

An intraoral periapical radiograph revealed a vertical (intrabony) defect extending from the distal surface of 16 to the mesial surface of 17, reaching the middle third of the root length, while the lamina dura appeared intact elsewhere.

## Diagnosis:

Based on the clinical and radiographic findings, the case was diagnosed as localized chronic periodontitis with a vertical intrabony defect in relation to teeth 16 and 17.

## Treatment plan:

The treatment was carried out in three phases. The first phase involved non-surgical therapy, including scaling and root planing, followed by oral hygiene instruction and reinforcement. The patient was re-evaluated after four weeks, and persistent pocketing necessitated surgical intervention. The second phase consisted of open flap debridement and regenerative therapy using Bio-Oss xenograft with a collagen membrane. The third phase included supportive periodontal therapy with regular recall visits to maintain long-term results.

## Surgical procedure:

After obtaining informed consent, local anesthesia (2% lignocaine with adrenaline 1:80,000) was administered. A crevicular incision extending from 15 to 17 was made, and a full-thickness mucoperiosteal flap was reflected to expose the osseous defect. Thorough debridement and root planing were performed to eliminate granulation tissue and residual calculus. The intrabony defect was delineated and measured at approximately 7 mm from the alveolar crest to the base of the defect. Bio-Oss granules (Bio-Oss®; Geistlich Pharma AG, Switzerland) were gently packed into the defect to the crest level, and GTR membrane (Bio-Gide®; Geistlich Pharma AG, Switzerland) was trimmed and adapted to completely cover the grafted site. The flap was repositioned and sutured with 4-0 black silk interrupted sutures.

## Post-operative care:

The patient was prescribed amoxicillin 500 mg three times daily for five days and a combination of ibuprofen and paracetamol three times daily for three days. Chlorhexidine mouth rinse (0.12%) was advised twice daily for two weeks, and mechanical plaque control at the surgical site was avoided for ten days. Sutures were removed after two weeks, and the healing was satisfactory.

## Follow-up and clinical outcome:

At 1 months, the site exhibited satisfactory healing. At 3 month, complete soft-tissue healing was achieved. By the 6-month, the gingiva appeared clinically healthy with no tooth mobility and probing pocket depth reduced to 3 mm. Radiographic evaluation at six months showed significant bone fill in the previously defective area, confirming successful periodontal regeneration and clinical stability.

## Discussion:

Periodontal regeneration aims to restore the lost or damaged supporting structures of teeth, including the alveolar bone, periodontal ligament and cementum, which are destroyed in periodontitis. Current regenerative treatments such as guided tissue regeneration (GTR), enamel matrix derivatives, bone grafts, and growth factor delivery seek to promote the formation of new, functional periodontal tissues. Despite advances, predictability of these outcomes varies, and newer approaches involving tissue engineering, stem cell therapy, and biomaterial scaffolds are being explored to improve regenerative success [2]. Periodontal regeneration is a critical therapeutic goal aimed at restoring the tooth-supporting structures lost due to periodontitis. Regenerative periodontal therapy involves techniques and biomaterials that promote the formation of new cementum, periodontal ligament, and alveolar bone to re-establish the original architecture and function of the periodontium. The use of xenograft bone substitutes like demineralized bovine bone mineral (Bio-Oss®) in combination with guided tissue regeneration (GTR) membranes (Bio-Gide®) has been well documented to facilitate periodontal wound healing by providing a scaffold for cell repopulation and maintaining space for regeneration [3]. Such combined regenerative approaches have shown promising clinical and radiographic outcomes in reducing periodontal pockets and enhancing bone fill in intrabony defects, thus improving tooth prognosis and long-term retention. This case exemplifies the practical application and effectiveness of xenograft-based regenerative therapy in localized chronic periodontitis. The successful management of periodontal intrabony defects depends on achieving new attachment formation and regeneration of lost alveolar bone. In the present case, the combination of Bio-Oss and a collagen membrane provided a biologically conducive environment for periodontal regeneration [4]. Bio-Oss acts as an osteoconductive scaffold that supports the ingrowth of new bone by allowing migration of osteogenic cells from the adjacent bone surfaces. Its porous structure enhances vascularization and space maintenance, which are critical for successful regeneration. The resorbable collagen membrane, on the other hand, ensures compartmentalization by excluding epithelial and connective tissue cells, allowing selective repopulation by periodontal ligament and bone cells [2]. Previous studies have demonstrated that xenografts, such as Bio-Oss, result in significant clinical attachment gain and reduction in probing depth compared to open flap debridement alone. Zhang *et al.* reported that Bio-Oss Collagen, a bioactive xenograft, facilitates periodontal regeneration through its structural similarity to human cancellous bone, osteoinductive capacity, and superior moldability, providing excellent space maintenance [3]. Similarly, Li *et al.* demonstrated that regenerative therapy in intrabony defects results in predictable attachment gain and long-term tooth retention when performed under strict maintenance programs [5]. The defect morphology plays a crucial role in regenerative success. Three-walled defects and contained lesions exhibit greater potential for regeneration due to the presence of bone walls that provide a natural scaffold. In this case, the defect morphology and localized nature favored successful graft stabilization and bone fill. Moreover, patient-related factors such as optimal oral hygiene, compliance, and regular recall visit significantly contributed to the positive clinical outcome [6]. Biomaterials like Bio-Oss have the advantage of slow resorption, maintaining volume stability during the healing process. Histological studies have shown that Bio-Oss particles become integrated within the newly formed bone without eliciting foreign-body reactions. The use of a collagen membrane enhances wound stabilization and accelerates early healing due to its biocompatibility and hemostatic properties. The synergistic use of these materials, therefore, promotes a favorable biological environment for the regeneration of lost periodontal structures [7]. The importance of patient motivation and supportive periodontal therapy cannot be overstated. As demonstrated by Gissler, Kroeger (2025), even teeth with an initially poor prognosis can be maintained over several years if the patient adheres to a strict recall and maintenance schedule [8]. In the present case, consistent follow-up and reinforcement of oral hygiene measures ensured long-term stability and prevented recurrence of periodontal destruction. Within the limitations of this case, it can be concluded that xenograft-based regenerative therapy provides a viable and predictable alternative to extraction or resective treatment in localized intrabony defects. The clinical and radiographic improvements observed affirm the role of Bio-Oss and collagen membrane in promoting periodontal regeneration and preserving natural dentition [9].

## Conclusion:

Periodontal regeneration using Bio-Oss and a collagen membrane represents a predictable and effective treatment option for localized vertical intrabony defects associated with chronic periodontitis. The present case underscores the significance of proper case selection, precise surgical technique, and patient compliance. With optimal maintenance and regular recall visits, regenerative therapy can achieve long-term stability and restoration of periodontal health.

## Patient perspective and consent:

The patient expressed satisfaction with the aesthetic and functional outcome and reported no postoperative discomfort or recurrence of symptoms. Written informed consent was obtained for both the treatment procedure and publication of this case report and accompanying images, with assurance of patient anonymity.

## References

[1] https://www.ncbi.nlm.nih.gov/books/NBK554590/.

[2] Badarau-Suster AM (2025). Dentistry Journal..

[3] Zhang C (2020). BMJ opens..

[4] Zagade H (2022). International Journal of Frontiers in Biology and Pharmacy Research..

[5] Li X (2023). Int J Implant Dent..

[6] Bud E (2025). Dentistry Journal..

[7] Zhang T (2025). Materials Today Bio.

[8] Gissler B (2025). Oral Health Prev Dent..

[9] Wang J (2024). J Appl Oral Sci..

